# Unraveling Biogeographic Boundaries Within the Sierra Madre Oriental, México: An Endemicity Analysis Using a Taxonomically Diverse Dataset

**DOI:** 10.1002/ece3.70779

**Published:** 2025-01-17

**Authors:** Irene Goyenechea Mayer‐Goyenechea, Gustavo Montiel‐Canales, Juan Márquez, Claudia T. Hornung‐Leoni, Jesús M. Castillo‐Cerón, Norma L. Manríquez‐Morán

**Affiliations:** ^1^ Centro de Investigaciones Biológicas (CIB) Universidad Autónoma del Estado de Hidalgo Mineral de la Reforma Hidalgo Mexico; ^2^ Centro de Estudios Científicos y Tecnológicos no 16 Hidalgo, Instituto Politécnico Nacional, Pachuca: Distrito de Educación, Salud Tecnología e Innovación San Agustín Tlaxiaca Hidalgo Mexico

**Keywords:** areas of endemism, multi‐taxa, NDM/VNDM, provinces, regionalization, áreas de endemismo, multi‐taxón, NDM/VNDM, provincias, regionalización

## Abstract

The Sierra Madre Oriental (SMO) is a significant mountain range and one of Mexico's 14 biogeographical provinces. Its delimitation has been debated. This study aims to analyze the distribution of plants, beetles, odonates, amphibians, reptiles, and mammals using an endemicity analysis to identify endemism areas and confirm the SMO's biogeographical units. Georeferenced data for 326 species distributed in the Sierra Madre Oriental were compiled using QGIS software, and an endemicity analysis (EA) was carried out with NDM‐VNDM to evaluate taxon distribution congruence in predefined grids. Different grid sizes and specific parameters were used to identify areas of endemism, with an Endemicity Index (EI) assigned to measure the consistency of these areas. Six main areas of endemism (EA) were identified: two in the northern region and four in the southern region of the SMO. These areas are supported by several taxa, except mammals, which did not significantly contribute to the identified AEs. The study suggests new boundaries within the SMO, establishing the Rio Verde as the natural barrier in the north rather than the Moctezuma River. The multi‐taxonomic analysis supports dividing the SMO into two subprovinces, proposing a new delimitation based on the distribution of species with different dispersal capacities. This new regionalization can be useful for prioritizing conservation areas and designing more effective strategies. Future research should include more distribution data of mammals and birds to strengthen these results and better define the subprovinces and biogeographical districts of the SMO.

## Graphical abstract

We provide boundaries of the Sierra Madre Oriental in Mexico through a multi‐taxa endemicity analysis.

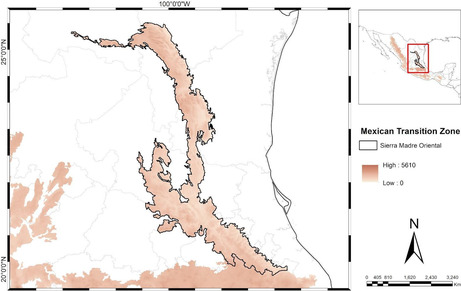

## RESUMEN

La Sierra Madre Oriental (SMO) es una importante cadena montañosa y una de las 14 provincias biogeográficas de México. Su delimitación ha sido objeto de debate. Este estudio tiene como objetivo analizar la distribución de plantas, escarabajos, odonatos, anfibios, reptiles y mamíferos utilizando un análisis de endemicidad para identificar áreas de endemismo y confirmar las unidades biogeográficas de la SMO. Se recopilaron datos georreferenciados para 326 especies distribuidas en la Sierra Madre Oriental utilizando el software QGIS, y se realizó un análisis de endemicidad (EA) con NDM‐VNDM para evaluar la congruencia de la distribución de los taxones en cuadrículas predefinidas. Se utilizaron diferentes tamaños de cuadrícula y parámetros específicos para identificar áreas de endemismo, asignando un Índice de Endemicidad (EI) para medir la consistencia de estas áreas. Se identificaron seis áreas principales de endemismo (AEs): dos en la región norte y cuatro en la región sur de la SMO. Estas áreas están respaldadas por varios taxones, excepto los mamíferos, que no contribuyeron significativamente a las AEs identificadas. El estudio sugiere nuevos límites dentro de la SMO, estableciendo el Río Verde como la barrera natural en el norte en lugar del Río Moctezuma. El análisis multi‐taxón apoya la división de la SMO en dos subprovincias, proponiendo una nueva delimitación basada en la distribución de especies con diferentes capacidades de dispersión. Esta nueva regionalización puede ser útil para priorizar áreas de conservación y diseñar estrategias más efectivas. Las investigaciones futuras deberían incluir más datos de distribución de mamíferos y aves para fortalecer estos resultados y definir mejor las subprovincias y distritos biogeográficos de la SMO.

## 1 Introduction

The Sierra Madre Oriental (SMO) is one of the 14 biogeographical provinces in Mexico and a significant mountain range within the Mexican Transition Zone (Morrone, Escalante, and Rodríguez‐Tapia 2017). It has been the subject of several proposals for its biogeographic delimitation which address physiographic, geological and biological aspects.

According to Espinosa, Aguilar, and Ocegueda (2004), this study considers it a natural biogeographical unit, given the solid foundation supporting this notion. However, differences in the exact definition of its boundaries and subdivisions persist, highlighting the complexity and richness of this region in terms of biogeographical and biodiversity. Several authors have proposed subprovinces and districts. Using the distribution of endemic species from various biological groups, Espinosa, Aguilar, and Ocegueda (2004), Espinosa‐Organista et al. (2008) and Morrone (2014a, 2014b, 2017, 2019) suggest dividing the SMO into two subprovinces separated by the Moctezuma River: Septentrional (north) and Meridional (south), each divided into two districts: Saltillo‐Parras and Sierra Gorda (west of the Septentrional subprovince) and Potosí and Zacualtipán (east of the Meridional subprovince) (Morrone 2020).

Other researchers conducted studies in the Sierra Madre Oriental (SMO) using siphonapterans (Gutiérrez‐Velázquez and Acosta‐Gutiérrez 2004) and mammals (León‐Paniagua et al. 2004) and agreed on dividing the SMO into two portions. Meanwhile, Navarro et al. (2004), through an analysis of birds, concluded that it could be divided into three zones: North, Center, and South. In contrast, Márquez and Morrone (2004), analyzing coleotperans suggested that the SMO could be divided into two provinces.

Morrone (2020) argues that the SMO is a biogeographic province composed of two subprovinces and four districts, derived from the analysis of various taxa. The subprovinces are the Austro‐Oriental in the north and the Hidalguense in the south. The Pánuco basin coincides with the boundary of these subprovinces. The Austro‐Oriental subprovince is located north of the Moctezuma River, and comprises the Saltillo‐Parras and Potosí districts. The Saltillo‐Parras district corresponds to the Pliegue Saltillo‐Parras, the Sierras Transversales, and part of the Gran Sierra Plegada. The Potosí district corresponds to part of the Gran Sierra Plegada and the Llanuras Occidentales in the Sierra de Potosí. The Hidalguense subprovince is located south of the Moctezuma River and consists of the Sierra Gorda and Zacualtipán districts. The Sierra Gorda district is bounded by the Moctezuma and Verde rivers, while the Zacualtipán district is situated to the east, in the Sierra Norte de Puebla and the Sierra de Zacualtipán. However, research continues to refine and better understand the biogeographic complexity of this important region.

The identification of biotas or natural geographic areas can be derived from biological and geographical aspects, particularly the identification of biogeographic patterns (or areas of endemism), which allow for the generation of a biogeographic regionalization (Montiel‐Canales and Goyenechea‐Mayer Goyenechea 2021). This is a hierarchical system of delimiting natural areas that includes the categories of kingdom, region, domain, province, and district. These categories are obtained through the presence of endemic species or ecological communities within a specific region (Escalante 2009). Areas of endemism can be identified through methods such as Parsimony Analysis of Endemism (PAE), which uses cladograms to recognize patterns and preliminary relationships between areas of endemism, or Endemicity Analysis (EA), which employs optimization algorithms to recognize geographical congruence of species (Escalante 2015). The EA proposed by Szumik et al. (2002) and Szumik and Goloboff (2004) is a suitable method for establishing biogeographic regionalization through the analysis of taxon distribution across a grid and provides the calculation of endemicity indices for each taxon based on how well the distribution of taxa fits a given grid and collectively for the areas identified in the analysis. This method is one of the most widely used to identify areas of endemism (Noguera‐Urbano 2016) as it allows for biogeographic analyses without requiring knowledge of the phylogenetic relationships of the taxa (Szumik et al. 2012). So far, there are few studies conducted with multiple taxa due to limited access to verified geographic coordinates of different taxonomic groups that are part of a biogeographic province. This is the case for the Sierra Madre Oriental, where despite several biogeographic analyses having been conducted to define its boundaries or identify its biogeographic relationships, these have been achieved through the use of particular taxa. For example, Noguera‐Urbano and Escalante (2015) identified three areas of endemism within the Mexican Transition Zone using endemicity analysis through a study of Neotropical mammals. Santiago‐Alvarado, Rivas, and Luna‐Vega (2019) conducted a biogeographic analysis of the Mexican Transition Zone using plants and vertebrates. Villaseñor and Ortiz (2022) suggest using the neighboring biogeographic provinces of the SMO to facilitate its delimitation, as well as data from different organisms to recognize the subunits that comprise it. However, to date, no studies have been conducted in the SMO using different biological groups with varying dispersal capacities to more precisely identify the boundaries inside this biogeographic province. Therefore, the aim of this study was to analyze the distribution of plants, insects, amphibians, reptiles, and mammals through endemicity analysis to identify areas of endemism and confirm the biogeographic subprovinces and districts within the SMO.

## 2 Materials and Methods

### 2.1 Study Area and Species Distribution Data

**FIGURE 1:**
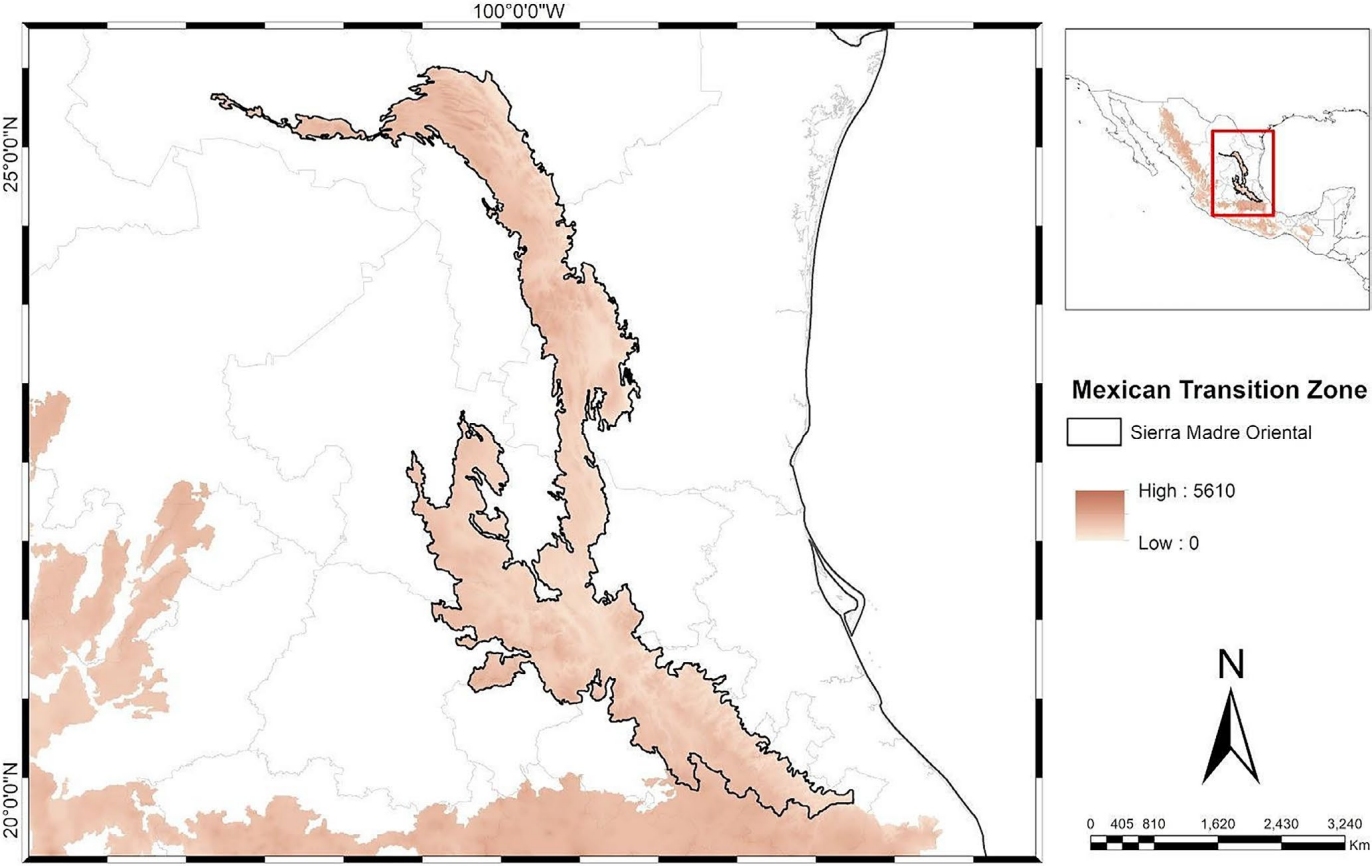
Location of the Sierra Madre Oriental in eastern Mexico. It includes the states of Coahuila, Guanajuato, Hidalgo, Nuevo León, Puebla, Querétaro, San Luis Potosí, Tamaulipas and Veracruz.

The SMO biogeographical province is the second largest mountain system in Mexico, spanning 1350 km^2^. This elongated and irregular mountain range stretches approximately 800 km in length, with a width varying between 80 and 100 km. Its geographic extent is defined longitudinally by the coordinates −102.3911 and −97.4050. It is part of the biotic assemblage east of the Mexican Transition Zone and extends across the states of Coahuila, Guanajuato, Hidalgo, Nuevo León, Puebla, Querétaro, San Luis Potosí, Tamaulipas, and Veracruz (Figure 1; Morrone 2020). A taxonomically diverse dataset was compiled that includes the geographic distribution of 326 species based on criteria that included restricted distribution ranges within the Sierra Madre Oriental (SMO) and representation across different taxonomic groups. Of these, 138 species (41%) are endemic to the SMO, highlighting their unique biogeographical significance, while 193 species (59%) are found both in the SMO and adjacent biogeographical provinces.

The selection of taxa was based on the congruence of distribution patterns, the availability of occurrence data, and prior knowledge about the species from previous biogeographical studies.

Dataset includes 326 species of plants (60 Cycadales, Ephedrales, Magnoliales, Pinales, Poales, and Saxifragales), insects (112 Coleoptera and Odonata), amphibians (34 Anura and Caudata), reptiles (47 Squamata and Testudines), and mammals (74 Carnivora, Cetartiodactyla, Cingulata, Lagomorpha, Didelphimorphia, Eulipotyphla, Pilosa, Primates, and Rodentia) (Table 1). The classification of the taxa was carried out following the APG IV (2016) for the orders of plants; Dijkstra et al. (2013) and Bouchard et al. (2011) for insects (odonates and beetles, respectively); Frost (2024) for amphibians; Uetz et al. (2024) for reptiles; and Burgin et al. (2018) for mammals.

**TABLE 1:** Plant and animal species included in the study.

Plantae	Cetartiodactyla
Cycadales	Cervidae
Zamiaceae	*Odocoileus virginianus*
*Ceratozamia hildae*	*Mazama americana*
*Ceratozamia kuesteriana*	Tayassuidae
*Ceratozamia mexicana*	*Pecari tajacu*
*Ceratozamia microstrobila*	*Tayassu pecari*
*Ceratozamia sabatoi*	Carnivora
*Ceratozamia zaragozae*	Canidae
*Dioon edule*	*Canis latrans*
*Zamia fischeri*	*Urocyon cinereoargenetus*
*Zamia loddigesii*	Felidae
*Dioon edule*	*Herpailurus yagouaroundi*
Ephedrales	*Leopardus pardalis*
Ephedraceae	*Leopardus wiedii*
*Ephedra antisyphilitica*	*Lynx rufus*
*Ephedra aspera*	*Panthera onca*
*Ephedra compacta*	*Puma concolor*
*Ephedra pedunculata*	Mephitidae
Araucariales	*Conepatus leuconotus*
Podocarpaceae	*Conepatus mesoleucus*
*Podocarpus reichei*	*Mephitis macroura*
Cupressales	*Mephitis mephitis*
Cupressaceae	*Spilogale putorius*
*Cupressus arizonica*	Mustelidae
*Cupressus lusitánica*	*Eira barbara*
*Juniperus deppeana*	*Galictis vittata*
*Juniperus flaccida*	*Lontra longicaudis*
*Juniperus monosperma*	*Mustela frenata*
*Juniperus montícola*	*Taxidea taxus*
*Taxodium mucronatum*	Procyonidae
Taxaceae	*Bassariscus astutus*
*Taxus globosa*	*Bassariscus sumichastri*
Pinales	*Nasua narica*
Pinaceae	*Potos flavus*
*Abies duranguensis*	*Procyon lotor*
*Abies guatemalensis*	Ursidae
*Abies religiosa*	*Ursus americanus*
*Abies vejarii*	Lagomorpha
*Picea engelmannii*	Leporidae
*Pinus arizonica*	*Lepus californicus*
*Pinus ayacahuite*	*Lepus callotis*
*Pinus cembroides*	*Sylvilagus audubonii*
*Pinus culminicola*	*Sylvilagus brasiliensis*
*Pinus engelmannii*	*Sylvilagus cunicularius*
*Pinus hartwegii*	*Sylvilagus floridanus*
*Pinus leiophylla*	Eulipotyphla
*Pinus montezumae*	Soricidae
*Pinus nelsonii*	*Cryptotis mexicanus*
*Pinus oocarpa*	*Cryptotis obscurus*
*Pinus patula*	*Cryptotis parvus*
*Pinus pinceana*	*Notiosorex crawfordi*
*Pinus pseudostrobus*	*Notiosorex villai*
*Pinus strobiformis*	*Sorex milleri*
*Pinus teocote*	*Sorex oreopolus*
*Pseudotsuga menziesii*	*Sorex saussurei*
Poales	*Sorex ventralis*
Bromeliaceae	Primates
*Aechmea mexicana*	Atelidae
*Catopsis sessiliflora*	*Ateles geoffroyi*
*Hechtia argéntea*	Rodentia
*Hechtia glomerata*	Cuniculidae
*Hechtia lepidophylla*	*Agouti paca*
*Bakerantha lundelliorum*	Erethizontidae
*Bakerantha hidalguensis*	*Coendou mexicanus*
*Pitcairnia longifolium*	Geomyidae
*Pitcairnia xanthocalyx*	*Cratogeomys castanops*
*Tillandsia arroyoensis*	*Cratogeomys goldmani*
*Tillandsia inopinata*	*Cratogeomys merriami*
*Tillandsia violacea*	*Cratogeomys neglectus*
Saxifragales	*Geomys tropicalis*
Crassulaceae	*Orthogeomys hispidus*
*Echeveria tolimanensis*	*Thomomys bottae*
*Echeveria trianthina*	*Thomomys umbrinus*
Animalia	Sciuridae
Insecta	*Ammospermophilus interpres*
Coleoptera	*Cynomys mexicanus*
Passalidae	*Glaucomys volans*
*Heliscus moroni*	*Ictidomys mexicanus*
*Heliscus tropicus*	*Otospermophilus variegatus*
*Heliscus vazquezae*	*Sciurus alleni*
*Odontotaenius zodiacus*	*Sciurus aureogaster*
*Oileus heros*	*Sciurus deppii*
*Oileus nonstriatus*	*Sciurus oculatus*
*Oileus rimator*	*Tamias bulleri*
*Petrejoides orizabae*	*Xerospermophilus perotensis*
*Proculejus hirtus*	*Xerospermophilus spilosoma*
*Proculejus sartorii*	Cingulata
*Pseudacanthus aztecus*	Dasypodidae
*Spurius halffteri*	*Dasypus novemcinctus*
*Verres corticicola*	Pilosa
*Vindex agnoscendus*	Cyclopedidae
*Yumtaax nebulosus*	*Tamandua mexicana*
Melolonthidae	Amphibia
*Amithao cavifrons*	Anura
*Chrysina macropus*	Bufonidae
*Chrysina peruviana*	*Incilius cristatus*
*Cotinis orientalis*	*Rhinella horribilis*
*Cyclocephala jalapensis*	Craugastoridae
*Cyclocephala lurida*	*Craugastor batrachylus*
*Cyclocephala sexpunctata*	*Craugastor decoratus*
*Dynastes hyllus*	*Craugastor mexicanus*
*Golofa pizarro*	Eleutherodactylidae
*Hemiphileurus dejeani*	*Eleutherodactylus cystignathoides*
*Hemiphileurus microps*	*Eleutherodactylus dennisi*
*Hoplia Asperula*	*Eleutherodactylus longipes*
*Hoplia squamifera*	*Eleutherodactylus verrucipes*
*Inca clathrata*	Hylidae
*Isonychus ocellatus*	*Agalychnis moreletii*
*Isonychus piperitus*	*Charadrahyla taeniopus*
*Macrodactylus fulvescens*	*Dryophytes arenicolor*
*Macrodactylus lineatocollis*	*Dryophytes eximius*
*Macropoidelimus mniszechi*	*Sarcohyla robertsorum*
*Macropoides nietoi*	*Sarcohyla charadricola*
*Paranomala sticticoptera*	*Scinax staufferi*
*Paranomala terroni*	*Smilisca baudini*
*Parisolea pallida*	Ranidae
*Pelidnota perplexa*	*Lithobates berlandieri*
*Phyllophaga rugipennis*	*Lithobates catesbeianus*
*Platycoelia humeralis*	*Lithobates johni*
*Plesiosternus setosus*	*Lithobates pueblae*
*Plusiotis aurofoveata*	Rhinophrynidae
*Plusiotis badeni*	*Rhinophrynus dorsalis*
*Plusiotis sallei*	Caudata
*Xyloryctes orientalis*	Ambystomatidae
Scarabaeidae	*Ambystoma velasci*
*Bolbelasmus rotundipennis*	Plethodontidae
*Canthon (Glaphyrocanthon) circulates*	*Aquiloeurycea cephalica*
*Coprophanaeus (Corpophanaeus) gilli*	*Aquiloeurycea galeanae*
*Coprophanaeus corythus*	*Aquiloeurycea scandens*
*Deltochilum (Calhyboma) mexicanum*	*Bolitoglossa platydactyla*
*Deltochilum scabriusculum*	*Chiropterotriton arboreus*
*Dichotomius satanas*	*Chiropterotriton chondrostega*
*Eurysternus magnus*	*Chiropterotriton magnipes*
*Eurysternus mexicanus*	*Chiropterotriton multidentatus*
*Geotrupes nebularum*	*Chiropterotriton priscus*
*Neoathyreus fissicornis*	Squamata
*Neoathyreus mixtus*	Anguidae
*Ontherus (Caelontherus) mexicanus*	*Abronia taeniata*
*Onthophagus incensus*	*Barisia imbricata*
*Onthophagus nasicornis*	*Celestus legnotus*
*Phanaeus (Phanaeus) amethystinus*	*Gerrhonotus infernalis*
Staphylinidae	Anolidae
*Agerodes amethystinus*	*Anolis naufragus*
*Belonuchus alternans*	*Anolis sericeus*
*Belonuchus bidens*	Corytophanidae
*Belonuchus colon*	*Laemanctus serratus*
*Belonuchus dichrous*	Crotaphytidae
*Belonuchus godmani*	*Crotaphytus collaris*
*Belonuchus jalappensis*	Dibamidae
*Belonuchus nigerrimus*	*Anelytropsis papillosus*
*Belonuchus platypterus*	Gekkonidae
*Chroaptomus flagrans*	*Hemidactylus frenatus*
*Eleusis bicolor*	Iguanidae
*Hesperus fasciatus*	*Ctenosaura acanthura*
*Homalolinus divisus*	Phrynosomatidae
*Homalolinus mexicanus*	*Sceloporus chaneyi*
*Homalolinus tlanchinolensis*	*Sceloporus goldmani*
*Leistotrophus versicolor*	*Sceloporus jarrovii*
*Misantlius carinulatus*	*Sceloporus olivaceus*
*Oligotergus fasciatus*	*Sceloporus scalaris*
*Oxyporus delgado*	*Sceloporus serrifer*
*Oxyporus flohri*	*Sceloporus variabilis*
*Peplomicrus mexicanus*	Scincidae
*Platydracus ferox*	*Plestiodon lynxe*
*Platydracus fuscomaculatus*	*Scincella silvicola*
*Platydracus gracilipes*	Teiidae
*Plociopterus fetialis*	*Aspidoscelis gularis*
*Scaphidium flohri*	Xantusiidae
*Scaphidium mexicanum*	*Lepidophyma gaigeae*
*Styngetus deyrollei*	*Lepidohyma micropholis*
*Thoracophorus sallaei*	*Lepidohyma occulor*
*Thyreocephalus unicolor*	*Lepidophyma pajapanensis*
*Xanthopygus rufipennis*	*Lepydophyma sylvaticum*
Odonata	Xenosauridae
Amphipterygidae	*Xenosaurus newmanorum*
*Amphipteryx agrioides*	*Xenosaurus platyceps*
Calopterygidae	Boidae
*Hetaerina capitalis*	*Boa imperator*
*Hetaerina infecta*	Colubridae
*Heteragrion tricellulare*	*Chersodromus rubriventris*
Coenagrionidae	*Pliocercus elapoides*
*Argia calida*	*Storeria hidalgoensis*
*Argia cuprea*	*Thamnophis exsul*
*Argia rudolphi*	*Tamnophis mendax*
*Ischnura posita*	Dipsadidae
*Mecistogaster modesta*	*Geophis latifrontalis*
*Protoneura cupida*	*Geophis multitorques*
*Pseudostigma aberrans*	*Rhadinaea gaigeae*
Gomphidae	*Rhadinaea marcellae*
*Erpetogomphus erici*	*Rhadinaea montana*
*Erpetogomphus liopeltis*	Elapidae
*Phyllogomphoides suasus*	*Micrurus diastema*
Lestidae	Leptotyphlopidae
*Archilestes regalis*	*Leptotyphlops dulcis*
Libellulidae	Viperidae
*Brechmorhoga vivax*	*Bothrops asper*
*Libellula herculea*	*Crotalus lepidus*
Platystictidae	*Crotalus molossus*
*Palaemnema paulicoba*	*Metlapilcoatlus nummifer*
Thaumatoneuridae	Testudines
*Paraphlebia zoe*	Emydidae
Mammalia	*Terrapene carolina*
Didelphimorphia	Kinosternidae
Didelphidae	*Kinosternon cruentatu*
*Didelphis marsupialis*	
*Didelphis virginiana*	
*Marmosa mexicana*	
*Philander opossum*	

Georeferenced records were visualized and analyzed using QGIS (QGIS Version 3.28) considering the regionalization of the SMO from Morrone (2020). Geographical data were verified through specialized literature and existing records combining field data of the species studied by the authors, databases (Museum of Zoology, UNAM; Institute of Biology, UNAM; Biological Collections of the Center for Biological Research, UAEH), and previous biogeographical studies of the SMO.

### 2.2 Endemicity Analysis (NDM‐VNDM)

Endemicity analysis (EA) identifies areas of endemism, which are geographic regions where the distribution ranges of two or more taxa overlap (Szumik and Goloboff 2004). This method is based on Platnick's (1991) theoretical concept of an area of endemism and provides a framework for identifying biogeographic regionalizations without requiring knowledge of the phylogenetic relationships of the taxa involved (Szumik et al. 2012). EA has become widely used to delineate such regions by evaluating taxon distributions across a defined grid and calculating endemicity indices based on the congruence of each taxon's distribution within the grid (Noguera‐Urbano 2016).

The criterion implemented in NDM/VNDM assigns an endemicity index to each endemic taxon (*e*; taxon endemicity index) depending on how closely its distribution aligns with a set of cells representing an area of endemism. Additionally, an area endemicity index (*E*) is calculated by summing the individual *e* indices of the taxa that comprise each area (Szumik and Goloboff 2004). A taxon reaches a maximum *e* value of “1” if it occupies every evaluated cell and is absent from the rest of the grid, whereas lower congruence yields a lower *e* value. The *E* value reflects the number of endemic taxa within the area: the more endemic taxa, the higher the *E* value (Szumik and Goloboff 2004).

A database of 15,678 geographical coordinates from 326 taxa distributed across the SMO was constructed.

The EA algorithm was executed using NDM‐VNDM v. 3.1 (Goloboff 2022). Given that the SMO is elongated and irregular, the grid size used can significantly influence the efficiency and accuracy of identifying areas of endemism. Using optimally sized grids maximizes the precision of sympatry comparisons among species. Employing different resolutions in the analyses allows for a more detailed and nuanced understanding of the spatial distribution of species. Smaller grids provide greater detail and accuracy for identifying areas of endemism with restricted geographic ranges, which may form biogeographic districts nested within larger regions. Therefore, conducting exploratory analyses with multiple grid sizes represents an ideal strategy to balance the extent of the areas analyzed with the resolution of the detected patterns (Casagranda, Roig‐Junent, and Szumik 2009). To analyze the effect of the spatial variable on the identification of areas of endemism exploratory analyses were conducted using various grid sizes (0.25° × 0.25°, 0.5° × 0.5°, 0.75° × 0.75°, and 1° × 1°) with the final analyses employing a 1° × 1° grid (15 columns × 19 rows) due to its ability to identify the most robustly supported areas. This grid was preferred based on both the results of the exploratory analyses conducted and existing literature, as it aligns with the findings of Pinilla‐Buitrago et al. (2018) who also utilized this grid size in their study of the Mexican Transition Zone. Although no definitive standard exists for determining the optimal grid size, this scale was found to balance resolution and reliability effectively.

The selected parameters included: (1) areas with a minimum *E* value of 2.0 and at least two endemic species per area were preserved (2) permutation of two grids per run with 100 replicates, (3) an EI value of 0.6 for endemic species, and (4) a 50% flexible consensus of unique species (Aagesen, Szumik, and Goloboff 2013).

Overlapping subsets of areas were permitted, retaining those with 50% unique species, and areas with a minimum *E* value of 2.0 and at least two endemic species per area were preserved, and the boundary proportion option was not applied.

Once the areas of endemism were identified, the distribution ranges of the taxa within each area were inspected to refine endemicity values. Taxa with an *e* value of 0.6 or higher, indicating a strong fit with the grid cells defining the area, were retained in the analysis; this value was set in NDM/VNDM (Set minimum species score) to filter out taxa that did not meet this requirement, recalculating the *E* values for the refined areas. A strict consensus was obtained based on a 50% overlap in endemic taxa among areas (Escalante et al. 2010). This consensus combined all areas sharing at least 50% of endemic taxa (Aagesen, Szumik, and Goloboff 2013). The list of endemic taxa and the calculated *E* and *e* values from NDM/VNDM were exported for describing the areas of endemism, with results then formatted for cartographic representation in QGIS.

## 3 Results

**FIGURE 2:**
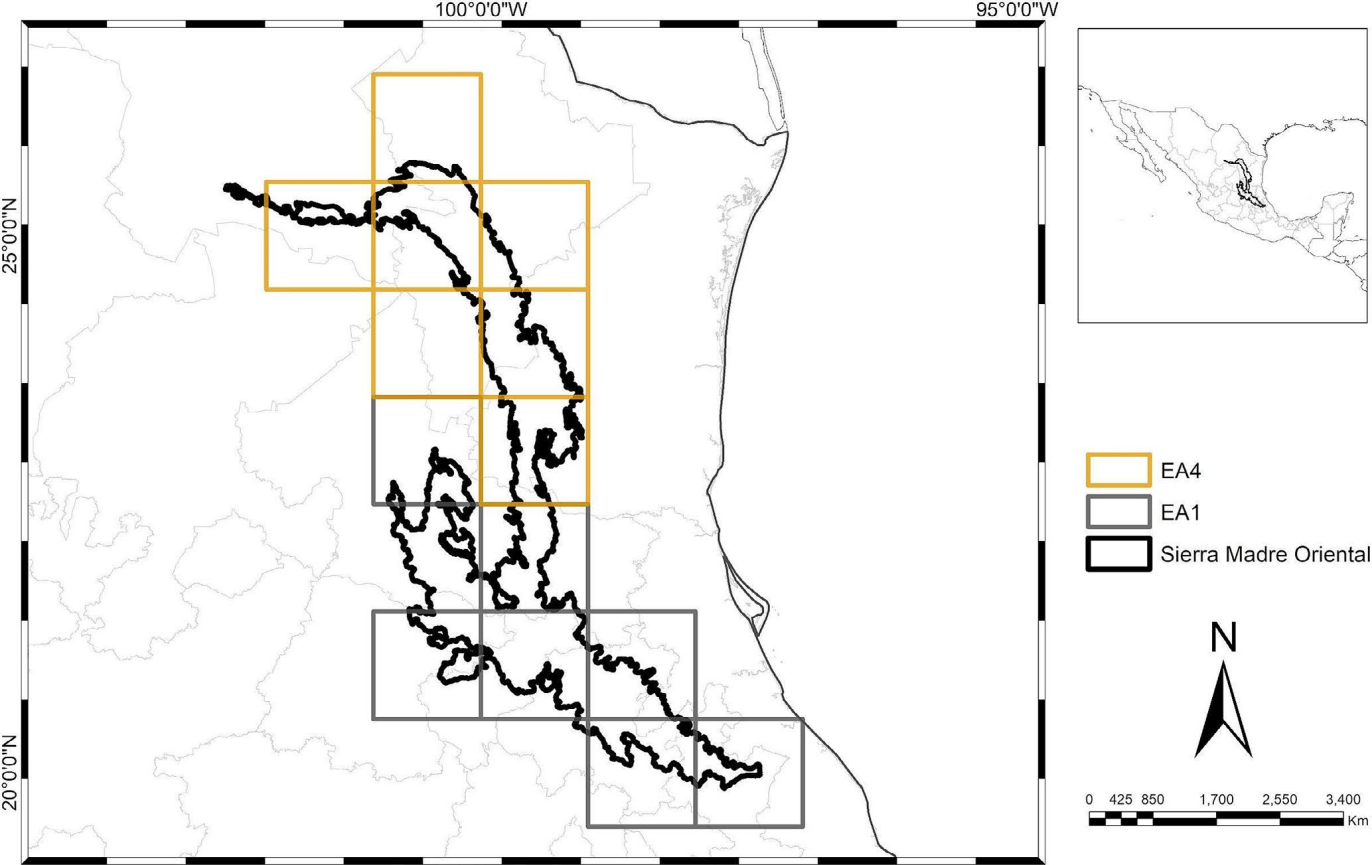
AE 1 and AE 4 show the SMO divided into two subprovinces, AE 1 (gray) in the south corresponding to the Hidalguense subprovince, and AE 4 (yellow) in the north corresponding to the Austral Oriental subprovince.

**FIGURE 3:**
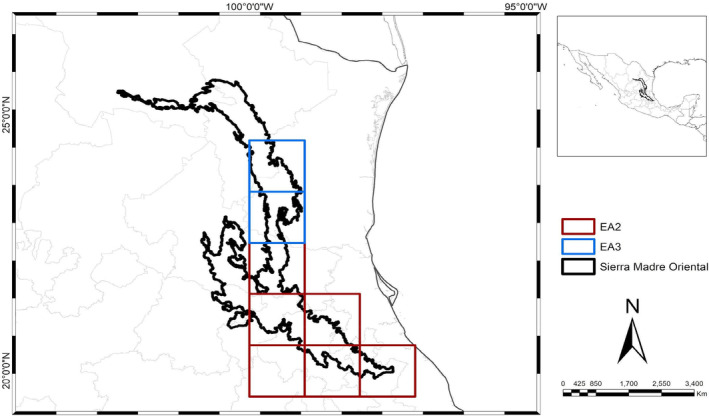
AE 2 and AE 3 show the SMO divided into two subprovinces, with AE 2 (red) in the south corresponding to the Hidalguense subprovince, and AE 3 (blue) in the north corresponding to the Potosí district.

**FIGURE 4:**
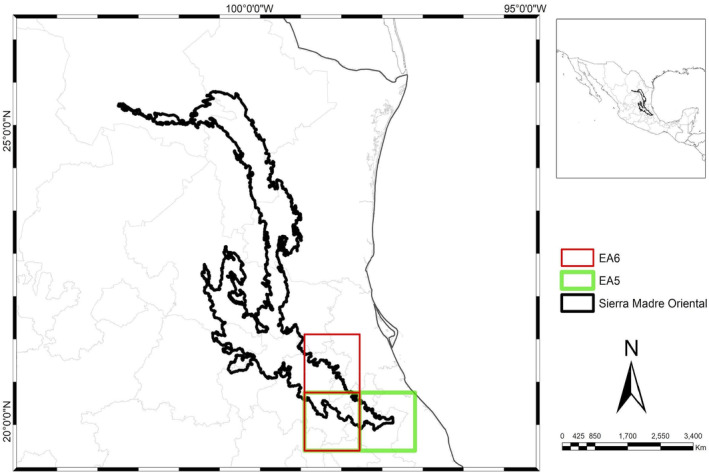
AE 5 and AE 6 in the south of the SMO, with AE 5 (green) corresponding to the Zacualtipán district, and AE 6 (red) corresponding to the Sierra Gorda district and part of the Zacualtipán district.

Sixteen areas of endemism were initially identified through the endemicity analysis. Areas that did not meet the minimum EI value of 2.0 or lacked sympatry in their distributions upon inspection were excluded. A consensus analysis of the remaining 16 areas resulted in six consensus areas of endemism (AE): two located in the northern region and four in the southern region of the SMO (Figures 2, 3, 4). These consensus areas displayed Endemicity Index (EI) values ranging from 2 to 74.07 and included a total of 113 endemic taxa. Detailed indices of total and taxon endemicity, as well as the endemic taxa associated with each area, are presented in Table 2.

**TABLE 2:** Location of the six Areas of Endemism (AE), the taxa comprising them, taxon endemicty index (*e*), and their Endemicity Index (EI) values within the SMO.

AE	Location	Comprising taxa	*e*	EI
AE 1	South SMO	*Argia calida*	0.600	5.6
		*Protoneura cupida*	0.625	
		*Libellula herculea*	0.750	
		*Chrysina macropus*	0.650	
		*Inca clathrata*	0.600	
		*Platydracus fuscomaculatus*	0.813	
		*Platydracus gracilipes*	0.625	
		*Eleutherodactylus verrucipes*	0.938	
AE 2	South SMO	*Magnolia rzedowskiana*	0.750	74.07
		*Echeveria trianthina*	0.750	
		*Catopsis sessiliflora*	0.833	
		*Hechtia argentea*	0.750	
		*Hechtia glomerata*	0.750	
		*Pitcairnia xanthocalyx*	0.833	
		*Tillandsia inopinata*	0.875	
		*Amphipteryx agrioides*	0.700	
		*Hetaerina capitalis*	0.750	
		*Hetaerina infecta*	0.833	
		*Heteragrion tricellulare*	0.750	
		*Paraphlebia zoe*	1.000	
		*Protoneura cupida*	0.700	
		*Argia rudolphi*	0.800	
		*Libellula herculea*	0.900	
		*Erpetogomphus erici*	0.833	
		*Heliscus vazquezae*	0.833	
		*Odontotaenius zodiacus*	1.000	
		*Oileus nonstriatus*	0.833	
		*Oileus rimator*	1.000	
		*Proculejus hirtus*	1.000	
		*Petrejoides orizabae*	0.600	
		*Proculejus sartorii*	0.900	
		*Pseudacanthus aztecus*	1.000	
		*Vindex agnoscendus*	0.833	
		*Spurius halffteri*	0.833	
		*Yumtaax nebulosus*	0.833	
		*Amithao cavifrons*	1.000	
		*Cyclocephala jalapensis*	0.875	
		*Cyclocephala sexpunctata*	1.000	
		*Golofa pizarro*	0.875	
		*Hemiphileurus microps*	0.833	
		*Hemiphileurus dejeani*	0.750	
		*Hoplia squamifera*	1.000	
		*Hoplia asperula*	0.833	
		*Isonychus ocellatus*	0.833	
		*Isonychus piperitus*	0.833	
		*Paranomala sticticoptera*	0.833	
		*Paranomala terroni*	0.833	
		*Platycoelia humeralis*	0.833	
		*Macropoidelimus mniszechi*	0.833	
		*Parisolea pallida*	1.000	
		*Macropoides nietoi*	0.833	
		*Plusiotis aurofoveata*	1.000	
		*Chrysina macropus*	0.714	
		*Plusiotis badeni*	0.833	
		*Neoathyreus fissicornis*	0.833	
		*Geotrupes nebularum*	0.833	
		*Deltochilum mexicanum*	1.000	
		*Deltochilum scabriusculum*	0.750	
		*Dichotomius satanas*	0.833	
		*Onthophagus nasicornis*	0.833	
		*Eurysternus magnus*	0.875	
		*Coprophanaeus gilli*	0.833	
		*Phanaeus amethystinus*	1.000	
		*Eleusis bicolor*	0.875	
		*Peplomicrus mexicanus*	0.750	
		*Oxyporus delgadoi*	0.833	
		*Oxyporus flohri*	0.750	
		*Scaphidium flohri*	0.750	
		*Misantlius carinulatus*	1.000	
		*Belonuchus alternans*	1.000	
		*Belonuchus bidens*	0.900	
		*Belonuchus colon*	1.000	
		*Belonuchus dichrous*	0.900	
		*Belonuchus jalapensis*	1.000	
		*Belonuchus godmani*	0.875	
		*Belonuchus nigerrimus*	0.875	
		*Belonuchus platypterus*	1.000	
		*Leistotrophus versicolor*	0.800	
		*Chroaptomus flagrans*	0.875	
		*Platydracus fuscomaculatus*	1.000	
		*Platydracus gracilipes*	0.700	
		*Plociopterus fetialis*	0.700	
		*Oligotergus fasciatus*	0.875	
		*Xanthopygus rufipennis*	0.750	
		*Agerodes amethystinus*	0.875	
		*Homalolinus mexicanus*	0.750	
		*Thyreocephalus unicolor*	1.000	
		*Sarcohyla charadricola*	1.000	
		*Lithobates pueblae*	0.750	
		*Chiropterotriton arboreus*	1.000	
		*Chiropterotriton magnipes*	0.750	
		*Lepidophyma occulor*	0.750	
		*Chersodromus rubriventris*	0.750	
		*Geophis multitorques*	0.600	
		*Rhadinaea marcellae*	0.833	
AE 3	North SMO	*Lepidophyma micropholis*	0.750	2.25
		*Xenosaurus platyceps*	0.750	
		*Thamnophis mendax*	0.750	
AE 4	North SMO	*Pinus culminicola*	1.000	6.45
		*Tillandsia arroyoensis*	1.000	
		*Aquiloeurycea galanae*	1.000	
		*Chiropterotriton priscus*	0.875	
		*Sceloporus chaneyi*	0.833	
		*Xenosaurus platyceps*	0.750	
		*Rhadinaea montana*	1.000	
AE 5	South SMO	*Heliscus vazquezae*	1.000	2.00
		*Geotrupes nebularum*	1.000	
AE 6	South SMO	*Catopsis sessiliflora*	1.000	6.00
		*Pitcairnia xanthocalyx*	1.000	
		*Oileus nonstriatus*	1.000	
		*Spurius halffteri*	1.000	
		*Vindex agnoscendus*	1.000	
		*Yumtaax nebulosus*	1.000	

*Note:* Plants: Magnoliales, Pinales, Poales, Saxifragales. Insects: Odonata, Coleoptera Amphibians: Anura, Caudata, Reptiles: Squamata.

AEs were recovered from different analyzed taxa except for mammals. Despite analyzing 73 mammal species, none significantly contributed to the AEs. The group of pines also contributed minimally, with only AE 4 comprising a pine species. In contrast, four of the six AEs included beetles. AE 1 is situated across the states of San Luis Potosí, Guanajuato, Querétaro, Hidalgo, Puebla, and Veracruz, representing the most extensive geographic area. The EI of this area is 5.6 and it is supported by the distribution of eight different taxa: four beetles, three odonates, and one amphibian. AE 2 spans San Luis Potosí, Querétaro, Hidalgo, Puebla, and Veracruz, The EI of this area is 74.07, comprising 87 species from all taxa except pines and mammals. AE 3 is located within Nuevo León and Tamaulipas and is supported by an EI of 2.25 and three reptile species, including two lizards and one snake. AE 4 encompasses parts of Nuevo León, Tamaulipas, Coahuila, and San Luis Potosí, an EI of 6.45, with congruent distributions of seven species from various taxa: one amphibian, three lizards, one snake, one pine, and one bromeliad. AE 5 covers Hidalgo, Puebla, and Veracruz, an EI of 2, supported by the presence of two beetle species. Finally, AE 6 is also located in Hidalgo, Puebla, and Veracruz, with an EI of 6 and is supported by six species: two bromeliads and four beetles. Some of these areas are supported by higher EI values, such as AE 2 (74.07). Likewise, there are areas comprised of fewer species whose distributions are strictly confined to the evaluated grid. These areas, with an EI value of 1, are also well‐supported, as exemplified by areas 5 and 6.

## 4 Discussion

Historically, the SMO has been accepted as having two subprovinces: Septentrional and Meridional (Morrone 2020), divided by the Moctezuma River, which is a natural barrier between the states of Querétaro, San Luis Potosí, and Hidalgo. This multi‐taxon analysis supports dividing the SMO into two subprovinces (Figure 2): the north subprovince based on AEs 3 and 4, and the south subprovince based on AEs 1, 2, 5, and 6 (Figure 3). However, the boundaries of the subprovinces are found north of the Verde River in San Luis Potosí, which acts as a natural barrier. The Verde River is more extensive than the previously proposed border, stretching from southern San Luis Potosí to Veracruz, aligning with the limits of the Karst Huasteco physiographic province and part of southern San Luis Potosí, to the Zacualtipán district (Ferrusquía‐Villafranca 1990).

The proposed boundaries result from an optimization analysis, used for the first time in the SMO, with unrelated phylogenetic taxa with different dispersal capacities, modifying Morrone's (2020) SMO division, who summarized the information of the studies carried out up to the year of its publication.

This southern division is supported by AE 1 (Table 1; Figure 2), and the congruent distribution of eight different taxa: three beetles, four odonates and one amphibian, representing the most extensive area geographically, including the Karst Huasteco and a portion of southern San Luis Potosí, corresponding to the Sierras and Llanuras Occidentales (Morrone 2020).

AEs 2, 5, and 6 are nested within AE 1. AE 2, supported by 87 species from the study groups except for pines and mammals, corresponds to the Karst Huasteco (Cervantes‐Zamora et al. 1990) and includes the Sierra Gorda district (Morrone 2014b), not recovered as a district. AEs 5 and 6 (Figure 4) represent smaller patterns in the southern SMO. AE 5 is supported by two beetle species, and AE 6 by six species (two bromeliads and four beetles), located in Hidalgo, Puebla, and Veracruz, aligning with the Zacualtipán district (Espinosa, Aguilar, and Ocegueda 2004).

The number of endemism areas in the southern SMO may be due to high species richness (e.g., amphibians and reptiles) and extensive data from all analyzed groups in this part of the SMO, suggesting a need for more records from the northern SMO. Medina‐Romero, Castillo‐Cerón, and Goyenechea (2016) showed a common biogeographic pattern recovered for different taxonomic groups with different vagility in the south of the SMO.

The northern SMO is supported by AE 4 (Table 1; Figure 2) and the congruent distribution of seven species from various taxa: two amphibians, two lizards, one snake, one pine, and one bromeliad, stretching from San Luis Potosí to Coahuila and Nuevo León, aligning with the Septentrional subprovince and the Gran Sierra Plegada and Sierras y Llanuras Occidentales physiographic provinces (Ferrusquía‐Villafranca 1990; Cervantes‐Zamora et al. 1990).

AE 3 (Figure 4) also reflects the northern SMO pattern, supported by three reptile species: two lizards and one snake, but is less extensive geographically than AE 4. AE 3 is located in southern Tamaulipas, aligning with the southern Gran Sierra Plegada (Morrone 2020). The Saltillo‐Parras district was not recovered as part of the northern AEs, possibly due to its small size or the presence of arid‐adapted species with limited records. Similarly, southeastern San Luis Potosí was not recovered within any endemism areas, possibly due to the biogeographical data from diverse biological groups not exclusive to this SMO portion or a lack of studies.

The proposed AE scheme for the SMO can serve as a starting point for conservation prioritization, establishing biological corridors, and designing effective conservation strategies, given the AEs' diverse, unrelated taxa with different dispersal capacities.

Future studies aiming to better define the subprovinces and districts should consider mammal species with more restricted distributions in the Sierra Madre Occidental (SMO). Although the shrew *Cryptotis obscura* , which is characteristic of the SMO, was included (Morrone 2020), no mammals were found to support the obtained area estimates. Likewise, the results obtained in this study could be strengthened by including distribution data for birds, more plant species, and members of the fungi kingdom. On the other hand, it should be taken into account that the number of areas of endemism identified in the southern SMO may be due to the region's high species richness and the large amount of field collection data available.

## Author Contributions

**Irene Goyenechea Mayer‐Goyenechea:** conceptualization (lead), data curation (supporting), formal analysis (equal), funding acquisition (equal), investigation (lead), methodology (equal), project administration (lead), supervision (equal), validation (lead), visualization (equal), writing – original draft (lead), writing – review and editing (lead). **Gustavo Montiel‐Canales:** conceptualization (equal), data curation (lead), formal analysis (equal), investigation (equal), methodology (equal), writing – original draft (equal), writing – review and editing (equal). **Juan Márquez:** conceptualization (supporting), data curation (equal), formal analysis (supporting), investigation (equal), methodology (supporting), writing – original draft (supporting), writing – review and editing (supporting). **Claudia T. Hornung‐Leoni:** conceptualization (equal), data curation (equal), investigation (equal), methodology (supporting), writing – original draft (equal), writing – review and editing (equal). **Jesús M. Castillo‐Cerón:** conceptualization (equal), data curation (equal), formal analysis (equal), methodology (equal), project administration (equal), writing – original draft (equal), writing – review and editing (equal). **Norma L. Manríquez‐Morán:** conceptualization (equal), data curation (equal), formal analysis (equal), investigation (equal), methodology (equal), writing – original draft (equal), writing – review and editing (equal).

## Conflicts of Interest

The authors declare no conflicts of interest.

## Supporting information


Data S1.

## Data Availability

The data that supports the findings of this study are available in the Supporting Information of this article.
